# Elimination of *Taenia solium* transmission to pigs in a field trial of the TSOL18 vaccine in Cameroon

**DOI:** 10.1016/j.ijpara.2010.01.006

**Published:** 2010-04

**Authors:** Emmanuel Assana, Craig T. Kyngdon, Charles G. Gauci, Stanny Geerts, Pierre Dorny, Redgi De Deken, Garry A. Anderson, André P. Zoli, Marshall W. Lightowlers

**Affiliations:** a Institute of Tropical Medicine, Department of Animal Health, Nationalestraat 155, B-2000 Antwerpen, Belgium; b Veterinary Clinical Centre, The University of Melbourne, 250 Princes Hwy, Werribee, Vic. 3030, Australia; c University of Dschang, Faculty of Agronomy and Agricultural Sciences, P.O. Box 222, Dschang, Cameroon

**Keywords:** *Taenia solium*, Vaccination, Pigs, TSOL18, Field trial, Cameroon

## Abstract

A pilot field trial of the TSOL18 vaccine was undertaken in Cameroon. Two hundred and forty, 2–3 month-old piglets were distributed to 114 individual households in pairs. Vaccinated animals received three immunisations with 200 μg TSOL18 plus 5 mg Quil A and 30 mg/kg oxfendazole at the time of the second immunisation. Necropsies were undertaken when the pigs were approximately 12 months of age. Viable *Taenia solium* cysticerci were identified in 20 control pigs (prevalence 19.6%); no cysticerci were found in any of the vaccinated animals (*P *< 0.0001). Combined application of TSOL18 vaccination and a single oxfendazole treatment in pigs may be a relatively simple and sustainable procedure that has the potential to control *T. solium* transmission in endemic areas and, indirectly, reduce the number of new cases of neurocysticercosis in humans.

## Graphical abstract

**Figure:**
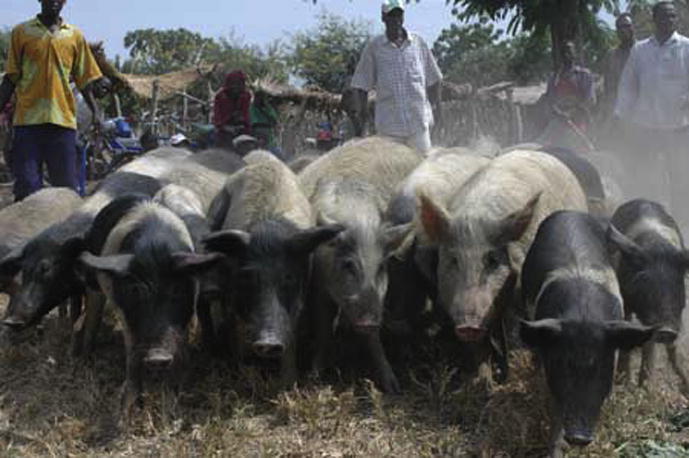

*Taenia solium* is a taeniid cestode parasite which is transmitted between humans and pigs, with pigs acting as the intermediate host. While humans are the obligatory definitive host for the parasite, they can also become infected with the metacestode life cycle stage and in such cases the parasite has a propensity to infect the brain, causing the disease neurocysticercosis. Neurocysticercosis is a debilitating disease prevalent in many parts of the developing world where pigs are allowed to roam free and where latrines are not available or not used (Garcia et al., 2003; Garcia and Del Brutto, 2005). The disease is also a burden in developed countries where immigrants infected with the adult worm can infect other citizens or arrive already suffering neurocysticercosis (Schantz et al., 1992).

Neurocysticercosis is a disease associated with poverty. Improvements in public sanitation in developed countries have led to the disappearance of *T. solium* transmission in many developed countries. Although the disease has been identified as having the potential to be eradicated on a global scale (Schantz et al., 1993), limitations in the usefulness of available tools have inhibited the initiation of widespread *T. solium* control programs and those that have been undertaken to date have achieved only a temporal decrease in disease transmission (Garcia and Del Brutto, 2005). A problem for *T. solium* control is that, although the adult tapeworm in humans is readily killed by treatment of infected persons with anthelmintics, in an environment where there are many pigs infected with the parasite new human tapeworm infections can be established and transmission of the disease can continue. Another potential control measure for *T. solium* is the treatment of infected pigs with anthelmintics to kill the muscle cysts. *Taenia solium* cysticerci in the muscles of pigs are killed following a single oral treatment with 30 mg/kg of the benzimidazole drug oxfendazole (Gonzales et al., 1996; Gonzalez et al., 1997; Sikasunge et al., 2008). However, there are two limitations to utilisation of this as an effective *T. solium* control measure. Following anthelmintic treatment, pigs that were not previously infected with *T. solium* remain susceptible to infection. Any infections acquired after anthelmintic treatment would be mature and capable of transmitting the disease following a period of approximately 6 weeks after the infection occurred. Unless the animals were slaughtered within this time window, anthelmintic treatment of pigs could not be relied upon to eliminate the potential for treated animals to transmit the disease. Coordinating anthelmintic treatment of pigs with their sale and slaughter, taking into consideration a withholding period for the animals after application of the chemical and the nature of most environments in which *T. solium* occurs, could be problematic. The second limitation to the use of anthelmintic treatment of pigs as a control measure for *T. solium* relates to the occurrence of substantial lesions in the meat of infected animals arising from the inflammatory reactions that occur in response to the anthelmintic-mediated death of cysticerci in the muscles. These lesions are not of concern for transmission of *T. solium* because the parasite is dead; however, they are unsightly and if present in significant numbers, make the meat unsuitable for sale. The lesions persist for as long as 6 months after pig treatment (Sikasunge et al., 2008).

Vaccination has been identified as a potentially valuable new tool for prevention of *T. solium* transmission (Lightowlers, 1999). While it may be potentially possible to vaccinate the human population against *T. solium*, a less expensive option is the vaccination of pigs to prevent the disease transmission thereby indirectly reducing the number of new human cases of neurocysticercosis. Several candidate vaccines have been developed and one has shown some promise in field trials (Huerta et al., 2001; Sciutto et al., 2007; Morales et al., 2008). The TSOL18 vaccine has proven to be the most effective vaccine against *T. solium*, with independent experimental vaccine trials carried out in Mexico, Peru, Cameroon and Honduras inducing 99.3–100% protection against an experimental challenge infection with *T. solium* eggs in pigs (Flisser et al., 2004; Gonzalez et al., 2005; Lightowlers, 2006).

The far-Northern region of Cameroon is highly endemic for *T. solium* (Assana et al., 2001, 2010; Zoli et al., 2003). In this region we undertook the first field evaluation of the TSOL18 vaccine in pigs against a natural exposure to the parasite acquired through the consumption of the faeces of humans infected with *T. solium* taeniasis.

The vaccine field trial was conducted from October 2008 to July 2009 in the Mayo-Danay administrative department of the Far North region of Cameroon. This region is characterised by having Sahelian-Sudanese climate (M’Biandoun et al., 2003) with a short rainy season (June–September) and a long dry season (October–May). The total human population is about 600,000 inhabitants. Predominant ethnic groups in the rural areas are animistic or Christian. Forty-one villages (Fig. 1) and 114 pig farms were selected on the basis of epidemiological information collected from a survey of the population and serological results on pigs (Assana et al., 2010). Coordinates of each pig farm were obtained by means of the Global Positioning Systems (GPS) using a handheld Garmin eMap device (Garmin, Olathe, USA). The region and village locations are depicted in Fig. 1 (Arcview GIS 3.2 (Environmental System Research Institute, Redlands, USA)).

The general strategy adopted for the trial was to vaccinate piglets at 2–3 months of age and give a booster immunisation 4 weeks later. At the time of the second immunisation, the pigs were given oxfendazole to kill any parasites that may already have established in the animals prior to vaccination. Controls were similarly treated with anthelmintic so that any effects of the procedure could be associated with vaccination per se. Controls were not vaccinated because multiple, independent previous experiments had indicated that vaccination of pigs with all components of the vaccine, other than the TSOL18 protein itself, did not prevent *T. solium* infection in pigs while the majority of pigs vaccinated with TSOL18 have no parasites following an experimental challenge infection (Flisser et al., 2004; Gonzalez et al., 2005). Vaccinated pigs were given an additional booster immunisation because at the time the trial was initiated there was insufficient information available to predict whether two immunisations would have been sufficient to protect the pigs for the duration of the trial.

Vaccinations started at the end of the rainy season after which pigs were allowed to roam free during the dry season. Typical farm practice was for the pigs to roam free around the village all day, return to their owners for feeding in the evening and be penned overnight. Two hundred and eighty, three month-old piglets which appeared to be without *T. solium* infection (determined using tongue examination) were purchased from amongst the village farms and the animals were distributed in matched pairs (one vaccinated, one control) in 114 selected pig farms. Piglets were doubly labelled using both a numbered earring and a microchip.

Sample sizes were calculated using Fisher’s exact test such that differences between control and vaccinated groups would be identified with 80% power where the vaccine showed 90% efficacy, the prevalence of cysticercosis in control animals was 10% and differences between the groups were detected at the *P *< 0.05 level. One hundred and six pairs were required, however, after allowing for possible losses a total of 240 pigs (120 pairs) were placed in the 114 selected farms, some of which were allocated two or three pairs of animals.

TSOL18 was expressed in *Escherichia coli*, purified and lyophilised prior to transport to Cameroon as described by Flisser et al. (2004) and Gonzalez et al. (2005) except that each dose of vaccine contained 200 μg TSOL18 plus 5 mg Quil A (Brenntag Biosector, Frederikssund, Denmark). Vaccines were supplied in 10 dose vials which were stored refrigerated over a period of up to 5 months prior to their application in the pigs. During field work lyophilized vaccine may have been exposed to ambient temperature (up to 40 °C) for periods up to 12 h. Rehydrated vaccines were used as soon as possible although these may have been exposed to ambient temperature for up to 2 h before injection. Pigs received their first immunisation approximately 2 weeks after they had been placed with their host farm. Pigs were 2–3 months of age at the time of the first immunisation with 1 ml vaccine injected intramuscularly near the base of the ear. A second, identical immunisation was given after an interval of approximately 4 weeks. At the time of the second injection all pigs, both vaccinated and controls, received oxfendazole (Dolthene® Merial) per os at a dose rate of 30 mg/kg which typically was approximately 15 ml per animal. Approximately 3 months after the second immunisation, vaccinated pigs received a third injection of vaccine i.e. when the animals were about 6 months of age. Blood samples were obtained from all animals via the jugular vein at the time of each treatment as well as 2 and 9 weeks after the second immunisation, 2 weeks after the third immunisation and at necropsy. Serum was separated and stored at −20 °C. Any animals that became unavailable during the trial were investigated to determine the circumstances that led to this happening. Farmers were paid 8000 FCFA (12 Euro) every month for hosting the animals during the trial.

Pigs were slaughtered between 12–13 months of age. The brain and musculature from half of the carcase, split longitudinally, were dissected from the carcase and the number and viability of cysticerci determined as described by Flisser et al. (2004). Where it was clear that there were many hundreds of cysticerci in a carcase, the total number in muscle was estimated by selecting two, 1 kg muscle samples from different regions of the carcase, counting carefully the number of viable and non-viable cysts in those samples to obtain a mean number per kg and estimating the total number in the remaining half of the carcase from the weight of the associated half carcase musculature. On every occasion the numbers in the entire heart and brain were determined precisely.

Serum antibody titres to TSOL18 vaccine in vaccinated pigs were obtained by ELISA using TSOL18 expressed as a maltose binding protein (MBP) fusion as described by Kyngdon et al. (2006). Plates (Nunc®, Polysorb) were incubated with 100 μg per well of TSOL18-MBP (5 μg/ml) in carbonate-bicarbonate buffer, pH 9.6 for 1 h at 37 °C and overnight at 4 °C. Antigen was discarded and wells incubated with 150 μl of PBS plus 2% new-born calf serum, 0.05% Tween20 (PBS-NBCS-T) for 1 h at 37 °C. After washing the plates, test sera were serially diluted from 1/100 to 1/102,400 in PBS-NBCS-T and plates incubated for 1 h at 37 °C. Bound specific antibody was detected after washing the plates and addition of 100 μl of rabbit anti-pig IgG peroxidase conjugate (SIGMA) at optimal dilution followed by adding the chromogen/substrate solution consisting of orthophenylene diamine (DAKO, #S2045) and H_2_O_2_ as per the manufacturer’s directions_._ Plates were incubated at 30 °C for 15 min, the reaction was stopped with 50 μl/well of 4 N H2SO4 and the absorbance measured at 492 nm (Multiscan EX, Termo Labsystems). Titres were calculated as the dilution of serum at which the O.D. equalled 0.5. Geometric mean titres were calculated; a titre of 50 was used in those cases where the O.D. at 1:100 of a vaccinated animal’s serum was greater than the mean + 2 S.D. of the value for control sera at 1:100, but less than O.D. 0.5.

McNemar’s test was used to compare the proportion of paired vaccinated pigs that were infected with the proportion of control pigs that were infected. Comparison of the number of cysts in vaccinated and control paired pigs was evaluated by Wilcoxon’s signed rank test. Spearman’s rank correlation coefficient was used to assess the association between the number of cysts in the muscles and the number of cysts in the brain in the 102 control pigs, and in the 20 control pigs which had cysts in the muscles. Stata/SE 11.0 for Windows (StataCorp, College Station, TX, USA) software was used and a two-sided *P*-value < 0.05 was considered to be statistically significant.

Over the duration of the trial, a total of 28 pigs became unavailable for follow-up, 10 from the vaccinated group and 18 control animals. The majority had been killed and consumed by a neighbour. No necropsy information was obtained from these animals. TSOL18 vaccinations were well tolerated by the animals with no adverse reactions noted or reported by the keepers of the pigs. At necropsy no lesions were identified that were likely to have been associated with the injections.

Twenty control animals (of 102 available at the end of the trial) were found to harbour cysticerci when autopsies were undertaken when the pigs were 12–13 months of age, representing a prevalence of 19.6%. Numbers of cysticerci ranged from three cysts to more than 37,000 cysts. Thirteen animals had an estimated parasite burden of ⩾1000 cysticerci. All infected animals were found to have viable cysticerci and 98% of the total number of cysts found were viable. The mean number of viable cysts in infected animals was 7142. Three animals were found to have non-viable cysticerci also. Fifteen animals had cysticerci in the brain as well as the muscle. All animals with cysts in the brain had viable cysts with 97% of the brain cysts being viable. Three animals had non-viable as well as viable cysts in the brain.

At the completion of the trial, 97 of the control/vaccinated paired pigs had both animals available for necropsy. Eighteen animals which were available for necropsy, comprising five control animals and 13 vaccinated animals, had their partner unavailable for necropsy. Data concerning all animals which were necropsied (97 pairs plus 18 unpaired animals) are detailed in Table 1. No cysticerci were found at necropsy anywhere in any of the vaccinated animals, including both those for which the paired control animals was necropsied as well as the 13 vaccinated animals for which the pair partner was not available for necropsy. Statistical comparison of the pairs of control and vaccinated animals (Table 1) showed a significant reduction in vaccinated animals in total cysts (*P *< 0.0001), viable cysts in muscles (*P *< 0.0001), total cysts in the brain (*P *= 0.0002) and viable cysts in the brain (*P *= 0.0002). There was a reduction in the prevalence of infection from 19.6% (19/97) in paired control pigs to 0% (0/97) in paired vaccinated pigs (*P *< 0.0001) (Table 2). Spearman’s correlation coefficient was 0.89 (95% Confidence Interval (CI) 0.84 to 0.92, *P *< 0.0001, *n *= 102) when assessing the association between total number of cysts in the muscles and total number of cysts in the brain. The correlation was 0.92 (95% CI 0.81 to 0.97, *P *< 0.0001) for the 20 pigs with cysts in the muscles. Cysts in the brain only occurred in pigs with at least 479 cysts in the muscles (Table 1).

Specific antibody titres against TSOL18 in vaccinated pigs are shown in Fig. 2. All vaccinated animals developed detectable titres of antibody following the initial immunisation. The titre was boosted to a geometric mean titre of 750 detected 2 weeks after the second immunisation. A single animal failed to respond to the second immunisation and a further 11 animals displayed relatively poor responses having titres ⩽300. The response following the third immunisation given 4 months after the first injection was pronounced, with specific antibody titres boosted to a geometric mean titre of 12,000. All those animals which responded poorly to the second immunisation responded well to the third injection. The animal which failed to respond to the second immunisation had an anti-TSOL18 titre of 26,000 after the third immunisation. With the exception of a single animal, all pigs had titres after the third immunisation that were grater than the titre seen after the second immunisation. At the time the animals were necropsied, all vaccinated animals remained seropositive, with titres ranging from 160 to 4000.

Vaccination with TSOL18 prevented any detectable infection with *T. solium* in pigs raised in circumstances where there was a 20% prevalence of infection in unvaccinated animals. This level of protection is consistent with the findings of several previous vaccine trials which were carried out under controlled conditions against an experimental challenge infection (Flisser et al., 2004; Gonzalez et al., 2005; Lightowlers, 2006; Cai et al., 2008). When applied as two immunisations approximately 1 month apart, the vaccine induces complete or almost complete protection against a challenge infection given within a few weeks of the second immunisation. The duration of protection has not been defined in experimental challenge trials, however, data from the field trial detailed here indicates that two immunisations in young pigs, together with a booster immunisation given when the animals are 6–7 months of age, was sufficient to protect the animals through until the age at which they are generally slaughtered for consumption (12–14 months). While it is unclear whether the third immunisation was required in order to maintain immunity to the end of the trial, the antibody response of the pigs to TSOL18 was boosted to a substantially greater level following the third immunisation than seen after the second injection, suggesting that this may have enhanced and prolonged the level of protection. Further field trials involving different groups given one, two or three immunisations would be required in order to determine the minimum as well as the optimal vaccine schedule in order to achieve a useful level of protection from infection.

All pigs in the trial were given a single treatment with oxfendazole at the time the vaccinated animals received their second immunisation. The purpose of this treatment in the vaccinated group of pigs was to eliminate any parasites that may already be present in the muscles prior to the animals being rendered immune to subsequent parasite challenge following vaccination. The TSOL18 vaccine utilises an antigen which is present only in the oncosphere and immediate post-oncospheral stages in the parasite’s development (Gauci et al., 2006) and, although the vaccine has not specifically been tested for its effects on post-oncospheral parasites, it is not anticipated to have any effect on established cysticerci. Control animals were also given oxfendazole treatment so as to allow any differences between the vaccinated and control groups to be assigned specifically and uniquely to the TSOL18 vaccine. It is likely that a proportion of the piglets had already been infected with cysticerci prior to their treatment with oxfendazole and that the overall prevalence of *T. solium* infection of pigs in this region exceeds the 19.6% detected in the control animals which had received oxfendazole at 3–4 months of age. Many unvaccinated animals harboured heavy burdens of infection suggesting that they had directly eaten faeces of a tapeworm carrier. In the Mayo-Danay region of far North Cameroon 90% of pigs are free roaming and more than 40% of houses that keep pigs do not have latrines (Assana et al., 2010). The limited available data suggests that *T. solium* infection is hyperendemic in northern Cameroon (Assana et al., 2001; Zoli et al., 2003).

In Cameroon as well as many other regions of the world, neurocysticercosis is a significant cause of human morbidity and mortality. There is increasing interest in developing new disease control tools for *T. solium* and in defining control measures that could lead to elimination of the disease (Garcia et al., 2007). The vaccine field trial described here has found that a relatively simple procedure combining the TSOL18 vaccine with a single oxfendazole treatment has the capacity to be implemented as an effective measure to control transmission of *T. solium* through pigs. Implementation of this approach could potentially lead to a reduction in the number of human taeniasis cases and, thereby, reduction in the number of new human cases of neurocysticercosis. An advantage of using a combination vaccination plus chemotherapy approach to cysticercosis control in pigs, in comparison to using chemotherapy alone, is that use of the vaccine allows a sufficient period of time to elapse after chemotherapy for any lesions in the meat caused by necrotic cysticerci to be resolved prior to the animals being slaughtered for consumption. During this period, all animals (previously infected or otherwise) are protected against *T. solium* infection by the vaccine.

In this field trial the potential for transmission of *T. solium* was eliminated in the treated animals. TSOL18 vaccination plus chemotherapy in pigs may be a relatively sustainable procedure applicable on a wide scale. It could be anticipated that a combination of both vaccination/oxfendazole treatment of pigs together with anthelmintic treatment of the human population to eliminate the adult tapeworms, particularly when control procedures were first implemented, would have the greatest and most rapid impact on reducing the incidence of neurocysticercosis (Lightowlers, 1999).

The data presented here represent a proof-of-principal, demonstrating the potential of pig vaccination to control *T. solium* transmission; it may not present a protocol that would be readily acceptable for field use. At present, the TSOL18 vaccine is applied as a minimum of two intramuscular immunisations. Future improvements in the vaccine, in relation to minimizing the number of exposures to the recombinant antigen required to induce/maintain protection, and changing the method of delivery from parenteral to an oral route, would enhance the ease with which the vaccine could be applied in undertaking *T. solium* control.

## Figures and Tables

**Fig. 1:**
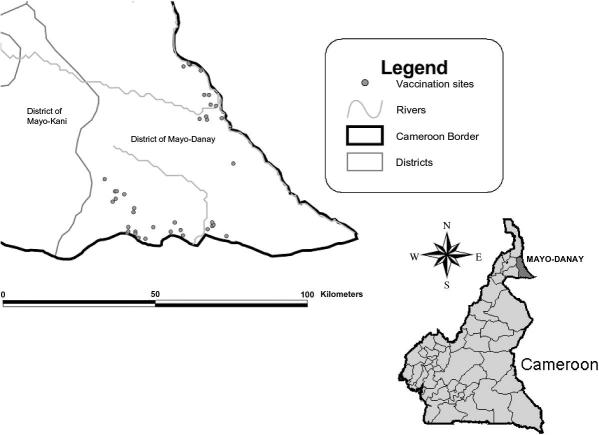
Location of the villages in the Mayo-Danay district of far north Cameroon in which host farms were located and pigs were kept during the field trial of the TSOL18 vaccine against *Taenia solium* infection.

**Fig. 2:**
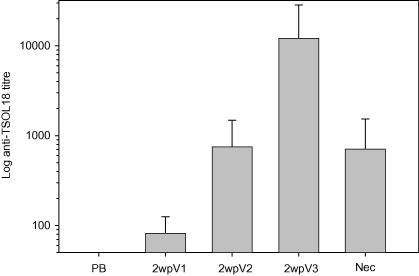
Anti-TSOL18 antibody titres (geometric mean ± S.D.) in vaccinated pigs following immunisation with TSOL18. Pigs received three immunisations and serum samples obtained prior to the first immunisation (PB) and 2 weeks after each immunisation (2wpV1, 2wpV2, 2wpV3) as well as at the time of necropsy (Nec). Antibody titres were determined against recombinant TSOL18 expressed as a maltose binding protein fusion with sera serially diluted from 1:100 and the titre calculated as the dilution at which the O.D. in ELISA equalled 0.5.

**Table 1:** Numbers of *Taenia solium* cysticerci in pairs of vaccinated and control pigs involved in a field trial of the TSOL18 vaccine in the Mayo-Danay district of Cameroon. Animals were distributed to different households as pairs comprising one control and one vaccinated animal. Cysticerci numbers were subsequently determined by detailed examination of carcase musculature and the brain at necropsy. The number of cysticerci was determined in half the carcase musculature plus the entire heart. Numbers for the entire brain are shown separately. Those animals for which both members of a pair were available for necropsy are shown as paired pigs while those where only one member of a pair was available for necropsy are shown as unpaired pigs.

Total cysts in control pig	Total cysts in vaccinated pig	Frequency	Muscle total cysts	Muscle viable cysts	Muscle non-viable cysts	Brain total cysts	Brain viable cysts	Brain non-viable cysts
*Paired pigs*								
0	0	78	0	0	0	0	0	0
3	0	1	3	3	0	0	0	0
6	0	1	6	6	0	0	0	0
10	0	1	10	10	0	0	0	0
14	0	1	14	14	0	0	0	0
100	0	1	100	100	0	0	0	0
484	0	1	479	479	0	5	5	0
784	0	1	781	781	0	3	3	0
1000	0	1	987	987	0	13	13	0
1500	0	1	1491	760	731	9	7	2
1536	0	1	1527	820	707	9	6	3
2052	0	1	2041	2041	0	11	11	0
2120	0	1	2116	2116	0	4	4	0
4000	0	1	3985	3985	0	15	15	0
10,560	0	1	10,543	10,543	0	17	17	0
14,976	0	1	14,955	14,955	0	21	21	0
15,000	0	1	14,981	14,981	0	19	19	0
18,368	0	1	18,343	18,343	0	25	25	0
32,000	0	1	31,978	31,978	0	22	22	0
37,100	0	1	37,080	37,080	0	20	20	0

*Unpaired pigs*								
0	−	4	0	0	0	0	0	0
3820	−	1	3812	2672	1140	8	7	1
−	0	13	0	0	0	0	0	0

**Table 2:** Summary of the prevalence of infection with *Taenia solium* in pairs of vaccinated and control pigs in a field trial of the TSOL18 vaccine in relation to the presence or absence of infection in the partner animal. Those animals for which data were available only from one member of a pair are shown as individual animals, with the associated pair indicated as unknown.

		*Taenia solium* infection status: vaccinates		
		+ve	−ve	Unknown
*Taenia solium* infection status: controls	+ve	0	19	1
	−ve	0	78	4
	Unknown	0	13	5

## References

[bib1] Assana, E., Amadou, F., Thys, E., Lightowlers, M.W., Zoli, A.P., Dorny, P., Geerts, S., 2010. Pig farming systems and porcine cysticercosis in the Far North region of Cameroon. J. Helminthol., in press. 10.1017/S0022149X10000167 PMC2965494 20334716

[bib2] Assana E., Zoli A., Sadou H.A., Nguekam, Vondou L., Pouedet M.S.R., Dorny P., Brand J.A., Geerts S. (2001). Prevalence of porcine cysticercosis in Mayo-Danay (North Cameroon) and Mayo-Kebbi (Southwest Chad). Rev. d’Elevage Med. Vet. Pays Trop..

[bib3] Cai X., Yuan G., Zheng Y., Luo X., Zhang S., Ding J., Jing Z., Lu C. (2008). Effective production and purification of the glycosylated TSOL18 antigen, which is protective against pig cysticercosis. Infect. Immun..

[bib4] Flisser A., Gauci C.G., Zoli A., Martinez-Ocana J., Garza-Rodriguez A., Dominguez-Alpizar J.L., Maravilla P., Rodriguez-Canul R., Avila G., Aguilar-Vega L., Kyngdon C., Geerts S., Lightowlers M.W. (2004). Induction of protection against porcine cysticercosis by vaccination with recombinant oncosphere antigens. Infect. Immun..

[bib5] Garcia H.H., Gonzalez A.E., Evans C.A., Gilman R.H. (2003). *Taenia solium* cysticercosis. Lancet.

[bib6] Garcia H.H., Del Brutto O.H. (2005). Neurocysticercosis: updated concepts about an old disease. Lancet Neurol..

[bib7] Garcia H.H., Gonzalez A.E., Del Brutto O.H., Tsang V.C., Llanos-Zavalaga F., Gonzalvez G., Romero J., Gilman R.H. (2007). Strategies for the elimination of taeniasis/cysticercosis. J. Neurol. Sci..

[bib8] Gauci C.G., Verastegui M.R., Gilman R.H., Lightowlers M.W. (2006). *Taenia solium* and *Taenia ovis*: stage-specific expression of the vaccine antigen genes, TSOL18, TSOL16, and homologues, in oncospheres. Exp. Parasitol..

[bib9] Gonzales A.E., Garcia H.H., Gilman R.H., Gavidia C.M., Tsang V.C., Bernal T., Falcon N., Romero M., Lopez-Urbina M.T. (1996). Effective, single-dose treatment or porcine cysticercosis with oxfendazole. Am. J. Trop. Med. Hyg..

[bib10] Gonzalez A.E., Falcon N., Gavidia C., Garcia H.H., Tsang V.C., Bernal T., Romero M., Gilman R.H. (1997). Treatment of porcine cysticercosis with oxfendazole: a dose-response trial. Vet. Rec..

[bib11] Gonzalez A.E., Gauci C.G., Barber D., Gilman R.H., Tsang V.C., Garcia H.H., Verastegui M., Lightowlers M.W. (2005). Vaccination of pigs to control human neurocysticercosis. Am. J. Trop. Med. Hyg..

[bib12] Huerta M., de Aluja A.S., Fragoso G., Toledo A., Villalobos N., Hernandez M., Gevorkian G., Acero G., Diaz A., Alvarez I., Avila R., Beltran C., Garcia G., Martinez J.J., Larralde C., Sciutto E. (2001). Synthetic peptide vaccine against *Taenia solium* pig cysticercosis: successful vaccination in a controlled field trial in rural Mexico. Vaccine.

[bib13] Kyngdon C.T., Gauci C.G., Gonzalez A.E., Flisser A., Zoli A., Read A.J., Martinez-Ocana J., Strugnell R.A., Lightowlers M.W. (2006). Antibody responses and epitope specificities to the Taenia solium cysticercosis vaccines TSOL18 and TSOL45–1A. Parasite Immunol..

[bib14] Lightowlers M.W. (1999). Eradication of *Taenia solium* cysticercosis: a role for vaccination of pigs. Int. J. Parasitol..

[bib15] Lightowlers M.W. (2006). Cestode vaccines: origins, current status and future prospects. Parasitology.

[bib16] M’Biandoun M., Guibert H., Olina J.P., Jamin Jean-Yves, Seiny Boukar L., Floret Christian (2003). Characterization of climate in four villages in the Soudano-sahelian area of North Cameroon and consequences for agriculture. Savanes africaines: des espaces en mutation, des acteurs face à de nouveaux défis. Actes du colloque, mai 2002.

[bib17] Morales J., Martinez J.J., Manoutcharian K., Hernandez M., Fleury A., Gevorkian G., Acero G., Blancas A., Toledo A., Cervantes J., Maza V., Quet F., Bonnabau H., de Aluja A.S., Fragoso G., Larralde C., Sciutto E. (2008). Inexpensive anti-cysticercosis vaccine: S3Pvac expressed in heat inactivated M13 filamentous phage proves effective against naturally acquired *Taenia solium* porcine cysticercosis. Vaccine.

[bib18] Schantz P.M., Moore A.C., Munoz J.L., Hartman B.J., Schaefer J.A., Aron A.M., Persaud D., Sarti E., Wilson M., Flisser A. (1992). Neurocysticercosis in an Orthodox Jewish community in New York City. N. Engl. J. Med..

[bib19] Schantz P.M., Cruz M., Sarti E., Pawlowski Z. (1993). Potential eradicability of taeniasis and cysticercosis. Bull. Pan. Am. Health Organ..

[bib20] Sciutto E., Morales J., Martinez J.J., Toledo A., Villalobos M.N., Cruz-Revilla C., Meneses G., Hernandez M., Diaz A., Rodarte L.F., Acero G., Gevorkian G., Manoutcharian K., Paniagua J., Fragoso G., Fleury A., Larralde R., De Aluja A.S., Larralde C. (2007). Further evaluation of the synthetic peptide vaccine S3Pvac against *Taenia solium* cysticercosis in pigs in an endemic town of Mexico. Parasitology.

[bib21] Sikasunge C.S., Johansen M.V., Willingham A.L., Leifsson P.S., Phiri I.K. (2008). *Taenia solium* porcine cysticercosis: viability of cysticerci and persistency of antibodies and cysticercal antigens after treatment with oxfendazole. Vet. Parasitol..

[bib22] Zoli A., Shey-Njila O., Assana E., Nguekam J.P., Dorny P., Brandt J., Geerts S. (2003). Regional status, epidemiology and impact of *Taenia solium* cysticercosis in Western and Central Africa. Acta Trop..

